# Prevalence and Associated Factors of Depression among Adults Suffering from Migraine in Spain

**DOI:** 10.3390/brainsci13121630

**Published:** 2023-11-24

**Authors:** Jesús Cebrino, Silvia Portero de la Cruz

**Affiliations:** 1 Department of Preventive Medicine and Public Health, Faculty of Medicine, University of Seville, Avda. Doctor Fedriani, S/N, 41009 Seville, Spain; jcebrino@us.es; 2 Department of Nursing, Pharmacology and Physiotherapy, Faculty of Medicine and Nursing, University of Córdoba, Avda. Menéndez Pidal, S/N, 14071 Córdoba, Spain; 3 Research Group GE10 Clinical and Epidemiological Research in Primary Care, Instituto Maimónides de Investigación Biomédica de Córdoba (IMIBIC), Hospital Universitario Reina Sofía, 14071 Córdoba, Spain

**Keywords:** depression, migraine disorders, population, trends

## Abstract

Considering the significance of migraine and the limited amount of research conducted on its association with depression in population-based studies in Spain, this study aimed to determine the prevalence and change of depression from 2017 to 2020 among adults with migraine in Spain and to analyze the sociodemographic and health-related variables linked to depression in migraine sufferers. A cross-sectional study on 5329 adults with migraine from the Spanish National Health Survey 2017 and the European Health Survey in Spain 2020 was performed. Binary logistic regression was used to examine the factors related to depression. A total of 26.32% of people with migraine suffered from depression. No significant changes in that prevalence between 2017 and 2020 were observed. The risk factors associated with depression included being between the ages of 25 and 44 (*p* = 0.018), being separated or divorced (*p* = 0.033), being unemployed (*p* < 0.001), not engaging in recreational physical activity (*p* = 0.016), perceiving one’s health as average, poor, or very poor (*p* < 0.001), experiencing moderate to severe pain in daily activities (*p* = 0.011, *p* = 0.004, *p* < 0.001, respectively), and having 1–2 or ≥3 chronic conditions (*p* = 0.003, *p* < 0.001, respectively). Conversely, being married (*p* = 0.001) and alcohol consumption (*p* = 0.007) were identified as protective factors.

## 1. Introduction

Migraine, a prevalent and disabling neurological disorder, affects around 1 billion people in the general population annually [1]. It is the second most prevalent neurological condition globally and makes a more substantial contribution to the burden of disability than other neurological disorders [2]. Migraine is described by recurrent episodes of throbbing, unilateral headaches, frequently accompanied by symptoms like photophobia or nausea, which can significantly impair daily functioning and may necessitate periods of bed rest [3,4].

Migraine and depression commonly coexist and are frequently encountered in clinical practice [5,6,7], with some studies confirming a bidirectional relationship between the two disorders [8,9]. In fact, depression is considered one of the most widespread psychiatric disorders in individuals with migraines [10], and its presence further increases the burden on migraine sufferers [11]. Evidence of the strong connection between migraine and depression dates back to 1990, when a study conducted in Zurich with young participants revealed this relationship [12], and it has recently been corroborated by some studies [13,14]. In this regard, migraine sufferers have a significantly higher likelihood of suffering depression compared to those without it [14], with studies indicating a 2.5-fold increased risk [15,16]. In this context, a meta-analysis indicated that the prevalence of depression in individuals with migraines varied between 8.6% and 47.9% [17]. Previous studies conducted in 2006 in Spain have also reported depression prevalence rates ranging from 14.25% to 42.05% among migraine sufferers [18,19,20].

Depression is a mood disorder marked by various symptoms, including diminished mood, loss of interest and pleasure, decreased motivation, reduced self-esteem, disrupted sleep and appetite, social withdrawal, and decreased productivity [21]. Lipton et al. [16] showed that people with comorbid migraine and depression experienced significant impacts on both their mental and physical health, resulting in a substantial reduction in their quality of life. The high prevalence of depression among individuals with migraines has a substantial impact on healthcare expenditures [22,23], in contrast to those with migraines alone [24].

According to recent global epidemiological data, the prevalence of migraine is projected at 14% [25], with a 12% prevalence in Spain [26]. Coupled with the scarcity of population-based studies on this disorder in Spain [18] and the psychological implications of this condition, particularly its association with depression, the unique value of the present study lies in its study of the relationship between a wide range of variables and depression, within a sizable sample of migraine sufferers living in Spain. The objectives of this study were, therefore, (i) to determine the prevalence and evolution over time of depression among adults with migraine in Spain, and (ii) to analyze the sociodemographic and health-related factors associated with depression in this population.

## 2. Materials and Methods

### 2.1. Design, Data Source, and Participants

We used secondary data from two sources to perform this cross-sectional study: the Spanish National Health Survey 2017 (SNHS 2017; October 2016 to October 2017) [27] and the European Health Survey in Spain 2020 (EHSS 2020; July 2019 to July 2020) [28]. These data were collected through personalized interviews conducted by the Ministry of Health, in collaboration with the National Institute of Statistics, among non-institutionalized individuals living primarily in familial residences in Spain. The interviews followed a stratified three-stage sampling method to gather the data, focusing on census sections first, households second, and, finally, a single adult from each household. Participants were sent a notification letter outlining the survey’s purpose and its voluntary, anonymous nature. They were informed that an expert interviewer would visit them. All subjects provided their informed consent. Further details regarding the methodology of the SNHS 2017 and EHSS 2020 can be found elsewhere [29,30].

**Figure 1:**
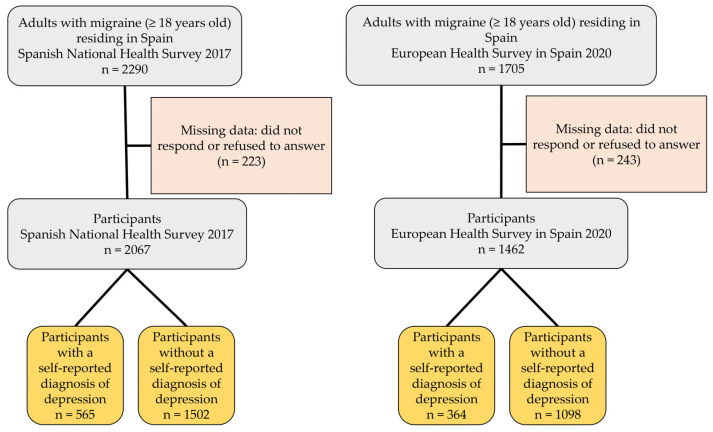
Flowchart of the study population (N = 3529).

For the data analysis, we included migraine sufferers aged ≥ 18 years who were residents in Spain during the survey years. We recognized individuals experiencing migraine as those who responded affirmatively to the query “Have you ever received a migraine diagnosis from a physician?”. The flowchart in Figure 1 illustrates the study population using data from the SNHS 2017 and EHSS 2020. From the initial pool of 3995 participants (SNHS 2017: n = 2290; EHSS 2020: n = 1705), 466 participants who either did not provide responses or declined to provide responses to the questions from the interview were excluded (SNHS 2017: n = 223; EHSS 2020: n = 243), which left a final sample of 3529 participants (SNHS 2017: n = 2067; EHSS 2020: n = 1462). Of these, 929 had a self-reported diagnosis of depression (SNHS 2017: n = 565; EHSS 2020: n = 364), while 2600 did not (SNHS 2017: n = 1502; EHSS 2020: n = 1098).

### 2.2. Variables

The dependent variable was defined as the “self-reported diagnosis of depression”, determined by the response to the question: “Have you been previously diagnosed with depression by a doctor?”. Participants responding “yes” were categorized as adults with a self-reported diagnosis of depression, while those responding “no” were categorized as adults without such a diagnosis.

The independent variables were divided into two categories:

(i) Sociodemographic characteristics: Year (2017, 2020); gender (women, men); civil status (single, married, widowed, separated, or divorced); educational attainment (without studies, primary, secondary or professional training, university); nationality (foreign, Spanish); employment situation (employed, unemployed) and socioeconomic class (social classes I and II, social classes III and IV, social classes V and VI) [31]. The age variable was categorized into four main life stages covering a significant part of the human lifespan: emerging adults (18–24 years old), young adults (25–44 years old), middle-aged adults (45–64 years old), and older adults (≥65 years). These stages were proposed by Arnett [32] for emerging adults and by Erikson [33] for the remaining age groups.

(ii) Health-related variables: recreational physical activity (yes, no) and number of days the respondent had walked in the last 7 days for at least 10 min (number of days); current smoker (yes, no); alcohol consumption in the past year (yes, no); self-perceived state of health (very good, good, average, poor, very poor); degree of pain experienced in the last 4 weeks (none, very mild, mild, moderate, severe, extreme); whether the pain affected their daily activities to a certain extent (none, a little, moderately, quite a bit, a lot); medicines taken for pain (yes, no) and the number of comorbidities (none, 1–2, ≥3). Finally, the body mass index (BMI) was computed using self-reported weight and height and categorized as: underweight, normal-weight, overweight, and obese [34].

### 2.3. Procedure and Ethical Considerations

The de-identified data we obtained are publicly available on the websites of the Spanish National Institute of Statistics and the Ministry of Health [29,30]. In accordance with Spanish law, Ethics Committee approval is not required for the use of these types of data.

### 2.4. Statistical Analysis

The qualitative variables were analyzed using counts and percentages, while the quantitative variables were analyzed using the arithmetic mean and standard deviation (SD). The normality of the variables was assessed through the Kolmogorov–Smirnov test. Means were compared using Student’s *t*-test. For contingency tables, the Chi-square test was utilized, and Fisher’s exact test was used when the expected frequencies exceeded 5. We used binary logistic regression to identify the characteristics related to the presence of depression, incorporating all variables in univariate tests that had a possible association with depression (*p* ≤ 0.15). Backward selection was used to exclude non-significant factors according to the Wald statistic’s likelihood. Crude and adjusted odds ratios (OR) with 95% confidence intervals were generated. The goodness of fit was examined using the Hosmer–Lemeshow test, with a *p*-value of 0.05 deemed significant. IBM SPSS Statistical Package version 26.0.0 (IBM Corp, Armonk, NY, USA), licensed to the University of Seville (Spain), was employed for the statistical analysis.

## 3. Results

### 3.1. Sociodemographic Characteristics and Health-Related Variables in Adults Suffering from Migraine in Spain by Self-Reported Diagnosis of Depression

The sample consisted of 3529 records of individuals with migraine aged ≥ 18 years residing in Spain. The majority of participants with migraine who had depression were women (*p* < 0.001), aged ≥ 65 years (*p* < 0.001), were unemployed (*p* < 0.001), belonged to socioeconomic classes V and VI (*p* < 0.001), had experienced an extreme level of pain in the last four weeks (*p* < 0.001), took medication for pain (*p* < 0.001), and had ≥ 3 comorbidities (*p* < 0.001) (Table 1).

### 3.2. Evolution over Time of the Prevalence of Depression in Adults with Migraine (2017–2020)

A total of 26.32% (n = 929) of people with migraine suffered from depression. Overall, the prevalence of depression did not vary from 2017 to 2020 (2017: 27.33%, 2020: 24.90%, *p* = 0.105).

### 3.3. Distribution of Adults with a Self-Reported Diagnosis of Depression and Migraine According to Sociodemographic Characteristics and Health-Related Variables from 2017 to 2020 in Spain

Overall, the percentage of participants with depression declined from 2017 to 2020 across different study variables, such as being aged 25–44 (*p* = 0.047), engaging in recreational physical activity (*p* = 0.019), being overweight (*p* = 0.007), having experienced no pain in the last 4 weeks (*p* = 0.025), the pain moderately affecting their daily activities to a certain extent (*p* = 0.035), and not consuming pain medications (*p* = 0.019). In contrast, experiencing an extreme degree of pain in the last 4 weeks (*p* = 0.011) and number of days the respondent walked in the last 7 days for at least 10 min (*p* < 0.001) increased among participants from 2017 to 2020 (Table 2).

### 3.4. Association between Depression and Sociodemographic Characteristics and Health-Related Variables in Adults with Migraine by Gender

In both genders, being unemployed (women: OR *=* 1.81, *p <* 0.001; men: OR *=* 1.99, *p <* 0.01), perceiving their health as average (women: OR *=* 2.20, *p <* 0.01; men: OR *=* 4.25, *p =* 0.02), poor (women: OR *=* 2.73, *p <* 0.01; men: OR *=* 6.37, *p <* 0.01), and very poor (women: OR *=* 3.27, *p <* 0.001; men: OR *=* 5.64, *p =* 0.02), as well as experiencing pain that moderately affected their daily activities (women: OR *=* 1.41, *p =* 0.03; men: OR *=* 1.32, *p =* 0.01), quite a bit (women: OR *=* 1.39, *p =* 0.04; men: OR *=* 2.05, *p =* 0.02), and a lot (women: OR *=* 2.07, *p <* 0.001; men: OR *=* 3.10, *p <* 0.01), and having 1–2 (women: OR *=* 1.55, *p =* 0.04; men: OR *=* 2.82, *p =* 0.03) and ≥ 3 chronic diseases (women: OR *=* 4.00, *p <* 0.001; men: OR *=* 5.50, *p <* 0.001) were all related with a greater likelihood of depression.

In women exclusively, being 25–44 years old (OR *=* 2.46, *p =* 0.03), separated or divorced (OR *=* 1.55, *p =* 0.03), and not engaging in recreational physical activity (OR *=* 1.32, *p =* 0.008) were associated with a higher probability of depression. In contrast, being married (OR *=* 0.69, *p =* 0.01) and having consumed alcohol in the past year (OR *=* 0.81, *p =* 0.04) were related with a lower likelihood of depression (Table 3).

### 3.5. Association between Depression and Sociodemographic Characteristics/Health-Related Variables in Adults with Migraine

Among adults with migraine, the likelihood of depression was greater among participants who were 25–44 years old (OR = 2.24; *p* = 0.018), were separated or divorced (OR = 1.43; *p* = 0.033), were unemployed (OR = 1.94; *p* < 0.001), did not engage in physical activity during leisure time (OR = 1.24; *p* = 0.016), perceived their health status as average, poor, and very poor (OR = 2.38, OR = 3.07, and OR = 3.39, respectively; *p* < 0.001), were affected moderately (OR = 1.40; *p* = 0.011), quite a bit (OR = 1.51; *p* = 0.004), and a lot (OR = 2.27; *p* < 0.001) by pain in their daily activities, and who had 1–2 (OR = 1.82; *p* = 0.003) and ≥3 (OR = 4.53; *p* < 0.001) chronic conditions. In contrast, being married (OR = 0.67; *p* = 0.001) and having consumed alcohol (OR = 0.78; *p* = 0.007) decreased the probability of depression (Table 4).

## 4. Discussion

### 4.1. Main Findings

The current study stands out by investigating the relationship between a broad spectrum of sociodemographic and health-related characteristics, and depression among people with migraine and depression residing in Spain.

In our study, 26.32% of individuals had depression, a percentage very similar to that showed by Fernández de las Peñas et al. [18], who obtained 25.71% in the same context. In other national studies conducted in Spain, the prevalence of people with migraines who suffered from depression, as reported by Jiménez-Sánchez et al. [20], was 14.25%, while Alonso-Blanco et al. [19] obtained 42.05%. On the other hand, the prevalence reported in other countries was higher in the United States and the United Kingdom [16,35], Taiwan [36], and Turkey [37], but lower in population-based studies in Canada [14], South Korea [38], and Pakistan [39].

Migraine usually affects young to middle-aged adults [40,41]. In our study, we found that the likelihood of depression was greater among individuals experiencing migraines in the age range of 25 to 44 years. Although depression is a common psychiatric disorder throughout adulthood, certain specific risk factors, such as comorbidities and pain, seem to exhibit a stronger association with depression during ages when its frequency is lower and less expected [42]. In fact, some studies showed that young women reported more risks for depression [43]. Additionally, a recent study by Bai et al. [44] suggested a decreasing trend in depression among young and middle-aged people, highlighting the evolving landscape of mental health in these age groups.

Migraine can cause a burden both at the individual level, such as limitations in performing activities at work and at home, and at the social level, such as indirect costs due to lost work time or unemployment [45]. In fact, according to a very recent study [46], people with migraine reported a higher likelihood of being unemployed due to their condition. Consistent with our findings, the probability of depression was higher in participants with migraine who were unemployed, in line with other studies [47,48]. In this regard, unemployment undermines mental health [49], leading to a greater percentage of depressive symptoms [50]. In relation to civil status, it was found that participants with migraine who were separated or divorced had a higher likelihood of experiencing depression. Going through one of the most stressful life events, such as separation or divorce, negatively impacts both physical and mental health [51,52], especially in women [53]. Additionally, Vladetić et al. [54] have demonstrated that subjects with headaches tend to evaluate experiences in a negative way and ending a marriage can precipitate depressive episodes [55]. Conversely, our finding that being married decreased the probability for experiencing depression in this population is not surprising, as the scientific literature suggests that being married can be a protective factor for mental health [56], particularly in relation to depression [57,58,59].

Regarding health-related factors, previous research has demonstrated an inverse association between physical activity and the prevalence of migraines [60,61,62]. In fact, a meta-analysis concluded that engaging in aerobic exercises improves migraine symptoms [63]. In that sense, another meta-analysis found that people who participated in substantial levels of physical exercise had a 17% reduction in the likelihood of experiencing depression compared to those with low levels of physical activity [64]. In contrast, another study observed a 51% increased risk of depression after two years in females who transitioned from an active to a sedentary lifestyle, in comparison to those who remained active [65]. According to Farris et al. [66], 80% of individuals who suffer from migraines intentionally avoid physical activity, and these researchers argue that this avoidance is due to the belief that physical activity will trigger or worsen migraines. Similarly, as pointed out by Denche-Zamorano et al. [67], not engaging in physical activity during leisure time has been related to a greater risk of depression in this population. As indicated by a systematic review of prospective studies [68], encouraging physical exercise may work as a mental health method for lowering the chance of developing depression in a community.

Alcohol should be regarded as a health hazard, given the potential for dependence and the elevated risks of injuries [69], diseases, and mortality associated with alcohol consumption [70]. In fact, alcoholic beverages have been identified as one of the most prevalent generator of migraine [71,72], with 27% of people with migraine reporting it according to a systematic review [73]. Similarly, there is broad agreement that risky alcohol consumption patterns are related to a higher incidence of depression [74,75]. The question asked both in the SNHS and EHSS was whether the participants had consumed alcoholic beverages in the past year, and the result obtained was that alcohol consumption decreased the probability of suffering from depression. Therefore, it is unknown from that question whether alcohol frequency is moderate or risky; although the scientific literature suggests that the predominant pattern is moderate intake, maintained by over half of the adult population [76]. However, in 2017, 75.2% of the adult Spanish population reported having consumed alcohol at least once in the last 12 months [77]. Overall, at the population level in Spain, there has been a decrease in alcohol consumption in recent years to pre-pandemic figures [78], with 16% being classified as risky alcohol consumption [79]. Based on all the above, numerous studies suggest that moderate alcohol consumption is related to a lower risk of depression [76,80,81,82,83]. In fact, it has been found that alcohol acts as a protective factor against depression in women, in line with Gea et al. [84]. This protective effect can be attributed to its ability to enhance mood and/or alleviate stress, given its common consumption during social activities [85].

The current study shows that Spanish people with migraine reported perceiving their health as average, poor, and very poor as a risk factor for depression, in line with similar findings from other studies [18,20,86]. Having a poor self-perceived health is associated with a lower quality of life and is linked to experiencing depression [87]. Similarly, experiencing pain was related to a higher likelihood of depression in individuals with migraine, consistent with other studies [88,89], supporting the notion that pain may be a symptom of depression [89,90,91,92]. In that sense, Ma et al. [93] found that when considering pain as a mediated variable, the effect of chronic illness on depression decreased, indicating that mediating variables can produce partially mediating effects. In our study, depression in those individuals whose pain moderately affected their daily activities decreased from 2017 to 2020. Lastly, it was found that the probability of depression was higher in participants with migraine who had multiple chronic diseases, as numerous studies have shown that both migraine and depression are often comorbid conditions, for example, with asthma, diabetes, or hypertension [94,95].

### 4.2. Strengths and Limitations

Although this study has several strengths, such as a large sample size, random population selection, and trained data collectors, there are also some possible limitations to consider. Firstly, because this was a cross-sectional study, causal relationships cannot be demonstrated for the observed associations. Therefore, future research employing longitudinal or experimental studies is needed to determine possible causal associations. Secondly, self-reported questions regarding diseases such as migraine or depression, among others, in terms of physician diagnosis were used. In this regard, there is no reliable way to verify whether participants actually suffer from the reported diseases, but the questions used by SNHS and EHSS are highly accurate. Thirdly, there are individuals who experience pain but do not seek medical diagnosis, which could result in underdiagnosed conditions, and thus, these individuals were not included in the current study. Fourthly, information regarding migraine characteristics such as severity, duration, or location was not collected. Fifthly, the information gathered during interviews may be susceptible to memory problems or people’ proclivity to make socially acceptable replies.

### 4.3. Implications for Research and Practice

The current study is notable for its examination of the correlation between depression and various sociodemographic and health-related factors among a representative sample of adults affected by migraines in Spain, and therefore, its findings warrant increased attention from health professionals and authorities. While in our study, no variation was observed between 2017 and 2020 regarding the prevalence of depression, perhaps because the EHSS only obtained data from July 2019 to July 2020, Hrytsenko et al. [96] found that people suffering from migraines experienced a significant decrease in depressive symptoms after the COVID-19 lockdown. This reduction may be associated with decreased work-related stress and, consequently, an overall improvement in the quality of life [97]. Considering the findings regarding health-related variables, it would be advisable to promote physical activity in this population, while also understanding the reasons for its avoidance, as the scientific literature recommends regular physical activity for migraine control [98] and confers protection against depression [64]. Moreover, it is important for healthcare professionals to be aware of the factors associated with depression in this group, and future interventions should focus on both pharmacological and non-pharmacological treatments to enhance their management.

## 5. Conclusions

The prevalence of depression among adults with migraine in Spain stands at 26.32%, which did not vary from 2017 to 2020. Furthermore, those more likely to experience depression are individuals with migraine who are aged 25–44, separated or divorced, unemployed, do not do physical activity during leisure time, perceive their health as average, poor, or very poor, experience moderate, quite a bit and a lot of pain in their daily activities, and have other comorbid conditions. Conversely, migraine sufferers who are married and those who consume alcohol are related to a lower likelihood of depression. Health professionals and authorities should take note of these associated characteristics in order to prevent depression and improve the management of this population.

## Figures and Tables

**Table 1:** Distribution of sociodemographic characteristics and health-related variables in adults suffering from migraine in Spain by self-reported diagnosis of depression (N = 3529).

Variables	Total n = 3529 n (%)	Adults with a Self-Reported Diagnosis of Depression n = 929 n (%)	Adults without a Self-Reported Diagnosis of Depression n = 2600 n (%)	*p*-Value
Gender				
Women	2598 (73.62)	728 (28.02)	1870 (71.98)	<0.001
Men	931 (26.38)	201 (21.59)	730 (78.41)	
Age groups				
18–24 years old	125 (3.54)	12 (9.60)	113 (90.40)	
25–44 years old	1000 (28.34)	183 (18.30)	817 (81.70)	<0.001
45–64 years old	1390 (39.39)	372 (26.76)	1018 (73.24)	
≥65 years old	1014 (28.73)	362 (35.70)	652 (64.30)	
Civil status				
Single	759 (21.51)	173 (22.79)	586 (77.21)	
Married	1992 (56.45)	449 (22.54)	1543 (77.46)	<0.001
Widowed	441 (12.49)	182 (41.27)	259 (58.73)	
Separated or divorced	337 (9.55)	125 (37.09)	212 (62.91)	
Educational attainment				
Without studies	451 (12.78)	180 (39.91)	271 (60.09)	
Primary	654 (18.53)	229 (35.02)	425 (64.98)	<0.001
Secondary or PT ^1^	1829 (51.83)	448 (24.49)	1381 (75.51)	
University	595 (16.86)	72 (12.10)	523 (87.90)	
Nationality				
Foreign	223 (6.32)	49 (21.97)	174 (78.03)	
Spanish	3306 (93.68)	880 (26.62)	2426 (73.38)	0.127
Employment situation				
Employed	1483 (42.02)	221 (14.90)	1262 (85.10)	
Unemployed	2046 (57.98)	708 (34.60)	1338 (65.40)	<0.001
Socioeconomic class				
Social classes I and II	562 (15.92)	84 (14.95)	478 (85.05)	
Social classes III and IV	1169 (33.13)	279 (23.87)	890 (76.13)	<0.001
Social classes V and VI	1798 (50.95)	566 (31.48)	1232 (68.52)	
Recreational physical activity				
Yes	2114 (59.90)	455 (21.52)	1659 (78.48)	
No	1415 (40.10)	474 (33.50)	941 (66.50)	<0.001
Current smoker				
No	2717 (76.99)	706 (25.98)	2011 (74.02)	0.401
Yes	812 (23.01)	223 (27.46)	589 (72.54)	
Alcohol consumption in the past year				
No	1466 (41.54)	517 (35.27)	949 (64.73)	<0.001
Yes	2063 (58.46)	412 (19.97)	1651 (80.03)	
Body Mass Index				
Underweight	93 (2.64)	23 (24.73)	70 (75.27)	
Normal weight	1486 (42.11)	306 (20.59)	1180 (79.41)	<0.001
Overweight	1218 (34.51)	342 (28.08)	876 (71.92)	
Obese	732 (20.74)	258 (35.25)	474 (64.75)	
Self-perceived state of health				
Very good	286 (8.10)	22 (7.69)	264 (92.31)	
Good	1307 (37.04)	133 (10.18)	1174 (89.82)	
Average	1239 (35.11)	417 (33.66)	822 (66.34)	<0.001
Poor	512 (14.51)	250 (48.83)	262 (51.17)	
Very poor	185 (5.24)	107 (57.84)	78 (42.16)	
Degree of pain experienced in the last 4 weeks				
None	906 (25.67)	111 (12.25)	795 (87.75)	
Very mild	225 (6.38)	43 (19.11)	182 (80.89)	
Mild	633 (17.94)	140 (22.12)	493 (77.88)	<0.001
Moderate	990 (28.05)	297 (30.00)	693 (70.00)	
Severe	644 (18.25)	269 (41.77)	375 (58.23)	
Extreme	131 (3.71)	69 (52.67)	62 (47.33)	
Whether the pain affected their daily activities to a certain extent				
None	1321 (37.43)	171 (12.94)	1150 (87.06)	
A little	707 (20.03)	166 (23.48)	541 (76.52)	
Moderately	675 (19.13)	216 (32.00)	459 (68.00)	<0.001
Quite a bit	511 (14.48)	208 (40.70)	303 (59.30)	
A lot	315 (8.93)	168 (53.33)	147 (46.67)	
Medicines taken for pain				
No	1193 (33.81)	208 (17.44)	985 (82.56)	<0.001
Yes	2336 (66.19)	721 (30.86)	1615 (69.14)	
Number of comorbidities				
None	602 (17.06)	36 (5.98)	566 (94.02)	
1–2	1096 (31.06)	149 (13.59)	947 (86.41)	<0.001
≥3	1831 (51.88)	744 (40.63)	1087 (59.37)	
**Variables**	**Mean (SD)**	**Mean (SD)**	**Mean (SD)**	***p*-Value**
Number of days the respondent had walked in the last 7 days for at least 10 min	3.99 (3.02)	3.41 (3.08)	4.20 (2.96)	<0.001

^1^ PT, professional training; SD, standard deviation.

**Table 2:** Distribution of adults with depression and migraine according to sociodemographic characteristics and health-related factors from 2017 to 2020 in Spain (n = 929).

Variables	2017 (n = 565) n (%)		2020 (n = 364) n (%)	*p*-Value
Gender				
Women	433 (28.69)		295 (27.09)	0.369
Men	132 (23.66)		69 (18.50)	0.061
Age groups				
18–24 years old	6 (8.96)		6 (10.34)	0.793
25–44 years old	121 (20.30)		62 (15.35)	0.047
45–64 years old	221 (27.42)		151 (25.86)	0.516
≥65 years old	217 (36.29)		145 (34.86)	0.640
Civil status				
Single	106 (24.65)		67 (20.36)	0.163
Married	283 (23.98)		166 (20.44)	0.063
Widowed	109 (41.44)		73 (41.01)	0.928
Separated or divorced	67 (34.54)		58 (40.56)	0.258
Educational attainment				
Without studies	108 (37.24)		72 (44.72)	0.120
Primary	152 (37.35)		77 (31.17)	0.109
Secondary or PT ^1^	260 (25.27)		188 (23.50)	0.383
University	45 (13.20)		27 (10.63)	0.342
Nationality				
Foreign	30 (22.39)		19 (21.35)	0.854
Spanish	535 (27.68)		345 (25.13)	0.102
Employment situation				
Employed	132 (15.71)		89 (13.84)	0.425
Unemployed	433 (35.29)		275 (33.58)	0.316
Socioeconomic class				
Social classes I and II	52 (16.05)		32 (13.45)	0.392
Social classes III and IV	158 (24.96)		121 (22.57)	0.340
Social classes V and VI	355 (31.98)		211 (30.67)	0.560
Recreational physical activity				
Yes	290 (23.27)		165 (19.01)	0.019
No	275 (33.50)		199 (33.50)	0.998
Current smoker				
No	428 (26.90)		278 (24.69)	0.195
Yes	137 (28.78)		86 (25.60)	0.316
Alcohol consumption in the past year				
No	330 (36.83)		187 (32.81)	0.116
Yes	235 (20.07)		177 (19.84)	0.899
Body Mass Index				
Underweight	12 (24.49)		11 (25.00)	0.955
Normal weight	183 (21.06)		123 (19.94)	0.598
Overweight	219 (31.02)		123 (24.02)	0.007
Obese	151 (34.09)		107 (37.02)	0.416
Self-perceived state of health				
Very good	10 (8.47)		12 (7.14)	0.677
Good	74 (9.74)		59 (10.79)	0.536
Average	261 (34.12)		156 (32.91)	0.662
Poor	148 (48.37)		102 (49.51)	0.799
Very poor	72 (61.02)		35 (52.24)	0.245
Degree of pain experienced in the last 4 weeks				
None	77 (14.26)		34 (9.29)	0.025
Very mild	24 (20.17)		19 (17.92)	0.669
Mild	83 (21.67)		57 (22.80)	0.738
Moderate	178 (31.62)		119 (27.87)	0.203
Severe	164 (43.73)		105 (39.03)	0.233
Extreme	39 (44.83)		30 (68.18)	0.011
Whether the pain affected their daily activities to a certain extent				
None	107 (14.06)		64 (11.43)	0.159
A little	95 (22.41)		71 (25.09)	0.410
Moderately	139 (35.19)		77 (27.50)	0.035
Quite a bit	121 (39.80)		87 (42.03)	0.615
A lot	103 (56.28)		65 (49.24)	0.216
Medicines taken for pain				
No	138 (19.57)		70 (14.34)	0.019
Yes	427 (31.35)		294 (30.18)	0.547
Number of comorbidities				
None	22 (6.85)		14 (4.98)	0.334
1–2	96 (14.81)		53 (11.83)	0.156
≥3	447 (40.71)		297 (40.52)	0.935
**Variables**	**2017** **Mean (SD)**	***p*-Value**	**2020** **Mean (SD)**	***p*-Value**
Number of days the respondent had walked in the last 7 days for at least 10 min	2.96 (3.14)	<0.001	4.12 (2.86)	<0.001

^1^ PT, professional training; SD, standard deviation.

**Table 3:** Logistic regression analysis for determinants of depression in adults with migraine by gender (N = 3529).

		Women (n = 2598)			Men (n = 931)	
Variables	OR (95% CI)	OR^a^ (95% CI)	*p*-Value	OR (95% CI)	OR^a^ (95% CI)	*p*-Value
Age groups						
18–24 years old	Reference	Reference		Reference		
25–44 years old	2.22 (1.05–4.71)	2.46 (1.09–5.55)	0.03	1.81 (0.61–5.34)		
45–64 years old	3.75 (1.79–7.88)	2.23 (0.98–5.08)	0.06	2.74 (0.95–7.89)		
≥65 years old	5.95 (2.83–12.50)	1.63 (0.70–5.55)	0.25	3.41 (1.17–9.94)		
Civil status						
Single	Reference	Reference		Reference		
Married	1.09 (0.85–1.38)	0.69 (0.51–0.92)	0.01	0.78 (0.54–1.11)		
Widowed	2.54 (1.91–3.39)	0.94 (0.65–1.37)	0.75	1.43 (0.68–3.00)		
Separated or divorced	2.28 (1.66–3.15)	1.55 (1.06–2.27)	0.03	1.20 (0.68–2.20)		
Educational attainment						
Without studies	Reference			Reference		
Primary	0.91 (0.69–1.21)			0.62 (0.36–1.04)		
Secondary or PT ^1^	0.49 (0.37–0.63)			0.50 (0.31–0.79)		
University	0.20 (0.14–0.28)			0.26 (0.13–0.50)		
Nationality						
Foreign	Reference			Reference		
Spanish	1.30 (0.90–1.88)			1.29 (0.64–2.59)		
Employment situation						
Employed	Reference			Reference	Reference	
Unemployed	2.75 (2.27–3.33)	1.81 (1.41–2.32)	<0.001	3.99 (2.76–5.73)	1.99 (1.32–2.99)	<0.01
Socioeconomic class						
Social classes I and II	Reference			Reference		
Social classes III and IV	1.81 (1.33–2.47)			1.70 (0.99–2.90)		
Social classes V and VI	2.76 (2.06–3.70)			2.15 (1.30–3.57)		
Recreational physical activity						
Yes	Reference			Reference		
No	1.85 (1.55–2.19)	1.32 (1.08–1.61)	0.008	1.70 (1.23–2.33)		
Current smoker						
No	Reference			Reference		
Yes	1.02 (0.83–1.25)			1.34 (0.95–1.89)		
Alcohol consumption in the past year						
No	Reference			Reference		
Yes	0.47 (0.40–0.56)	0.81 (0.66–0.98)	0.04	0.46 (0.33–0.64)		
Body Mass Index						
Underweight	Reference			Reference		
Normal weight	0.82 (0.49–1.36)			0.75 (0.15–3.70)		
Overweight	1.42 (0.85–2.38)			0.89 (0.18–4.38)		
Obese	1.75 (1.04–2.96)			1.55 (0.31–7.70)		
Self-perceived state of health						
Very good	Reference			Reference	Reference	
Good	1.24 (0.74–2.07)	0.87 (0.51–1.49)	0.611	2.09 (0.62–7.05)	1.61 (0.46–5.66)	0.460
Average	5.30 (3.25–8.66)	2.20 (1.29–3.74)	<0.01	1.58 (1.15–3.44)	4.25 (1.24–6.31)	0.02
Poor	9.79 (5.86–16.37)	2.73 (1.54–4.87)	<0.01	2.18 (1.11–4.38)	6.37 (1.79–22.72)	<0.01
Very poor	14.27 (8.00–25.44)	3.27 (1.71–6.27)	<0.001	3.26 (1.18–5.56)	5.64 (1.40–22.71)	0.02
Degree of pain experienced in the last 4 weeks						
None	Reference			Reference		
Very mild	1.66 (1.07–2.58)			1.55 (0.67–3.60)		
Mild	2.02 (1.47–2.77)			1.95 (1.13–3.37)		
Moderate	2.79 (2.11–3.69)			3.73 (2.32–6.00)		
Severe	4.46 (3.34–5.97)			7.34 (4.36–9.52)		
Extreme	7.36 (4.71–11.52)			8.79 (3.67–10.02)		
Whether the pain affected their daily activities to a certain extent						
None	Reference			Reference	Reference	
A little	1.95 (1.48–2.56)	1.16 (0.86–1.57)	0.323	2.29 (1.42–3.70)	1.38 (0.80–2.30)	0.26
Moderately	3.18 (2.44–4.14)	1.41 (1.04–1.90)	0.03	2.86 (1.80–4.55)	1.32 (1.10–2.21)	0.01
Quite a bit	4.17 (3.17–5.47)	1.39 (1.01–1.92)	0.04	5.99 (3.62–9.92)	2.05 (1.15–3.63)	0.02
A lot	7.22 (5.28–9.87)	2.07 (1.42–3.03)	<0.001	8.57 (4.88–11.36)	3.10 (1.62–5.94)	<0.01
Medicines taken for pain						
No	Reference			Reference		
Yes	2.08 (1.70–2.56)			2.00 (1.43–2.80)		
Number of comorbidities						
None	Reference			Reference	Reference	
1–2	2.15 (1.41–3.28)	1.55 (1.03–2.41)	0.04	4.03 (1.67–9.74)	2.82 (1.13–7.02)	0.03
≥3	9.44 (6.41–13.89)	4.00 (2.59–6.20)	<0.001	6.23 (2.18–9.02)	5.50 (2.27–13.32)	<0.001
Number of days the respondent had walked in the last 7 days for at least 10 min	0.91 (0.88–0.93)			0.96 (0.91–1.04)		

^1^ PT, professional training; OR, odds ratio; OR^a^, odds ratio adjusted for all sociodemographic characteristics and health-related variables; 95% CI, 95% confidence interval. Logistic regression for women: Hosmer–Lemeshow test χ^2^ = 8.69, *p*-value = 0.37; Nagelkerke’s R^2^ = 0.28; *p*-value < 0.001. Logistic regression for men: Hosmer–Lemeshow test χ^2^ = 5.25, *p*-value = 0.73; Nagelkerke’s R^2^ = 0.30; *p*-value < 0.001.

**Table 4:** Logistic regression analysis for determinants of depression in adults with migraine (N = 3529).

Variables	OR (95% CI)	OR^a^ (95% CI)	*p*-Value
Gender			
Men	Reference		
Women	1.41 (1.18–1.69)		
Age groups			
18–24 years old	Reference		
25–44 years old	2.11 (1.14–3.91)	2.24 (1.15–4.39)	0.018
45–64 years old	3.44 (1.88–6.31)	1.96 (1.00–3.85)	0.052
≥65 years old	5.23 (2.84–9.61)	1.39 (0.69–2.77)	0.355
Civil status			
Single	Reference		
Married	0.99 (0.81–1.20)	0.67 (0.53–0.86)	0.001
Widowed	2.38 (1.85–3.07)	0.94 (0.68–1.31)	0.712
Separated or divorced	2.00 (1.51–2.64)	1.43 (1.03–2.00)	0.033
Educational attainment			
Without studies	Reference		
Primary	0.81 (0.63–1.04)		
Secondary or PT ^1^	0.49 (0.39–0.61)		
University	0.21 (0.15–0.28)		
Nationality			
Foreign	Reference		
Spanish	1.29 (0.93–1.78)		
Employment situation			
Employed	Reference		
Unemployed	3.02 (2.55–3.58)	1.94 (1.56–2.41)	<0.001
Socioeconomic class			
Social classes I and II	Reference		
Social classes III and IV	1.78 (1.36–2.33)		
Social classes V and VI	2.61 (2.03–3.36)		
Recreational physical activity			
Yes	Reference		
No	1.84 (1.58–2.14)	1.24 (1.04–1.49)	0.016
Current smoker			
No	Reference		
Yes	1.08 (0.90–1.29)		
Alcohol consumption in the past year			
No	Reference		
Yes	0.46 (0.39–0.53)	0.78 (0.65–0.93)	0.007
Body Mass Index			
Underweight	Reference		
Normal weight	0.79 (0.48–1.29)		
Overweight	1.19 (0.73–1.93)		
Obese	1.66 (1.01–2.72)		
Self-perceived state of health			
Very good	Reference		
Good	1.36 (0.85–2.18)	0.95 (0.58–1.55)	0.843
Average	6.09 (3.88–9.55)	2.38 (1.46–3.86)	<0.001
Poor	11.45 (7.17–18.29)	3.07 (1.83–5.15)	<0.001
Very poor	16.46 (9.75–27.79)	3.39 (1.89–6.07)	<0.001
Degree of pain experienced in the last 4 weeks			
None	Reference		
Very mild	1.69 (1.15–2.49)		
Mild	2.03 (1.55–2.67)		
Moderate	3.07 (2.41–3.90)		
Severe	5.14 (3.99–6.62)		
Extreme	7.97 (5.36–11.85)		
Whether the pain affected their daily activities to a certain extent			
None	Reference		
A little	2.06 (1.63–2.61)	1.23 (0.95–1.60)	0.118
Moderately	3.16 (2.52–3.98)	1.40 (1.08–1.81)	0.011
Quite a bit	4.62 (3.64–5.86)	1.51 (1.14–2.00)	0.004
A lot	7.69 (5.85–10.10)	2.27 (1.63–3.15)	<0.001
Medicines taken for pain			
No	Reference		
Yes	2.11 (1.78–2.51)		
Number of comorbidities			
None	Reference		
1–2	2.47 (1.69–3.61)	1.82 (1.22–2.70)	0.003
≥3	10.76 (7.59–15.26)	4.53 (3.07–6.68)	<0.001
Number of days the respondent had walked in the last 7 days for at least 10 min	0.92 (0.90–0.94)		

^1^ PT, professional training; OR, odds ratio; OR^a^, odds ratio adjusted for all sociodemographic characteristics and health-related variables; 95% CI, 95% confidence interval. Hosmer–Lemeshow test χ^2^ = 17.55, *p*-value = 0.025; Nagelkerke’s R^2^ = 0.292; *p*-value < 0.001.

## Data Availability

The data presented in this study are available as Supplementary Materials (File S1: Research data).

## Supplementary Materials

The following supporting information can be downloaded at: https://www.mdpi.com/article/10.3390/brainsci13121630/s1, File S1: Research data.
